# Performance evaluation and comparative study of three 52-kW PV plants in India: a case study

**DOI:** 10.12688/f1000research.134731.1

**Published:** 2023-08-31

**Authors:** Divya Navamani Jayachandran, Boopathi Kadhirvel, Lavanya Anbazhagan, Geetha Anbazhagan, Pradeep Vishnuram, Reddy Prasad

**Affiliations:** 1 Department of Electrical and Electronics Engineering, SRM Institute of Science and Technology (Deemed to be University), Kattankulathur, Tamil Nadu, India; 2 National Institute of Wind Energy, Chennai, India; 3 Universiti Teknologi Brunei, Bandar Seri Begawan, Brunei-Muara District, Brunei

**Keywords:** 52-kW PV plant, energy yield, regression, prediction, Solar Energy.

## Abstract

Developing countries like India are rapidly transitioning from traditional energy sources to sustainable energy sources, due to the increase in demand and the depletion of fossil fuels. Grid-connected photovoltaic (PV) systems attract many investors, organizations, and institutions for deployment. This article studies and compares the performance evaluations of three 52-kW PV plants installed at an educational institution, SRMIST (SRM Institute of Science and Technology), in Tamil Nadu, India. This site receives an annual average temperature of 28.5°C and an average global horizontal irradiation of 160 kWh/m2/m. The prediction model for the 52-kW power plant is obtained using solar radiation, temperature, and wind speed. Linear regression model-based prediction equations are derived using the Minitab 16.2.1 software, and the results are compared with the real-time AC energy yield acquired from the three 52-kW plants for the year 2020. Furthermore, this 52-kW plant is designed using PVsyst V7.1.8 version software. The simulation results are compared with the energy yield from the plants in 2020 to identify the shortfall in the plant performance. The loss analysis for the plant is performed by obtaining the loss diagram from the PVsyst software. This study also proposes a methodology to study the commissioned PV plant’s performance and determine the interaction between variables such as direct and diffused solar radiations, air temperature, and wind speed for forecasting hourly produced power. This article will motivate researchers to analyze installed power plants using modern technical tools.

## Introduction

**Figure 1.**
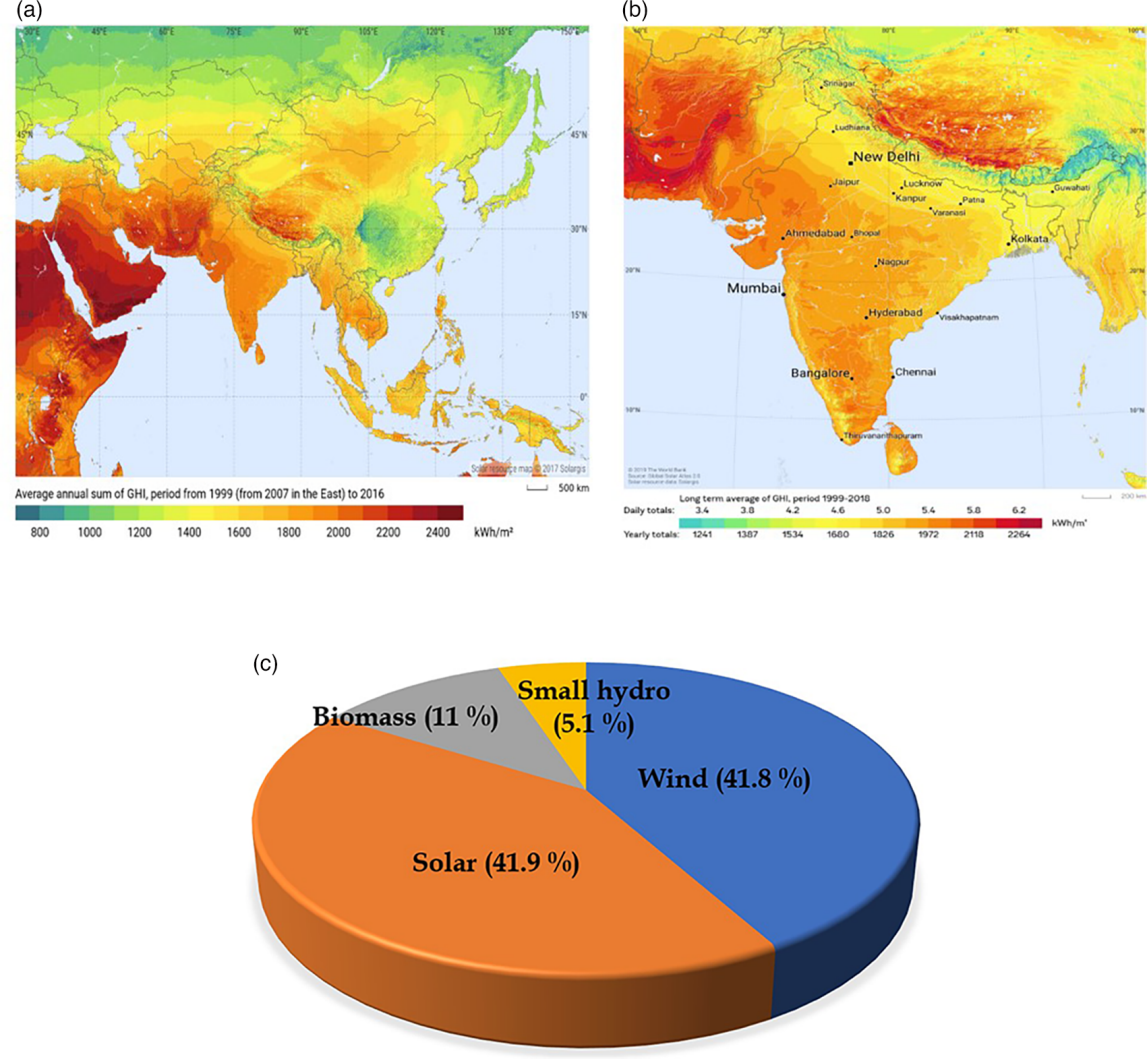
(a) Global solar radiation (GHI) of Asia; (b) Global solar radiation (GHI) of India. ^24^ Courtesy: Solargis maps; (c) Renewable energy (RE) share in India.

Solar energy has grown to be among the most popular sources of clean energy in recent years across many industries, and numerous studies are being conducted to improve its application and benefits. A continent like Asia has a higher potential for power generation from solar energy, as depicted in Figure 1(a). In this continent, developing country like India has vast potential, and their demand also increases with a population of nearly 140 crores. This power demand must be met through renewable power sources due to fossil fuel depletion. Since the country has higher global solar radiation, depicted in Figure 1(b), the country has set a target of 300 GW for solar energy by 2030. However, the country has already reached its installed capacity of 63 GW in March 2023, and the total installed capacity of renewable sources is depicted in Figure 1(c). Hence it is necessary to concentrate on this to reach the set target. Recently, many researchers have been concentrating on research based on several configurations in which solar photovoltaic (PV) systems can be installed, grid-connected PV and standalone PV, which may be designated as off-grid systems. ^1^ However, the installation capacity of both differs significantly as, through the years, it has been observed that a grid-connected PV system is much more developed than an off-grid system. There is another topology which is known as a Hybrid solar energy system. It can charge the system from the grid and solar PV directly, but these are expensive and are not usually preferred. Several studies are being conducted to improve its application in daily life and determine how its potential applications might be broadened. It has been observed that solar thermal collectors are utilized to turn energy into heat while also generating electricity with panels. ^2^ Some problems include variations in output energy due to changes in irradiance level. ^3^ As we know, PV modules are made from silicon cells, thus limiting their efficiency to significantly less. Therefore, it is essential to increase their efficiency so that more people can be ready to invest in this. ^4^ Solar insolation determines the sustainability and dependability of PV-based power generation systems; hence optimization is crucial to satisfy load demand. ^5^ These factors are crucial during system installation because the performance is also influenced by the environment, location, and plant varieties. ^6^

According to the IRENA report, even though many technologies exist, most governments concentrate on solar and wind, and major investments were made in PV and wind technology. ^7^ The government has recently initiated a series of efforts to deploy roof panels over various offices and organizations, which can assist in addressing the situation. ^8^^,^^9^ Sometimes, PV degradation may also lead to variation in series, shunt resistances, and decreased output power. ^10^ It can also diminish the impact of greenhouse gases brought by fossil fuels. PV energy is more affordable than any other sustainable energy source, and research has shown that it is incredibly profitable in rural regions. ^11^^,^^12^ At every stage of the global solar PV supply chain, China is currently by far the largest supplier; with a manufacturing capacity for PV modules of around 340 GW/year, it has more than twice the installed PV modules worldwide, with a manufacturing capacity utilization rate for solar components of between 40 and 50% in 2021. China directly supplies all markets, except North America, where import taxes on Chinese solar PV components have been imposed. However, Chinese businesses have been actively investing in production capacity in Southeast Asia to supply the area and export to the United States. ^13^ Many studies suggest the appropriate areas for implementing PV systems, but they could be more extensive. The utilized parameters and sites discussed in this research are identified as an outcome of the literature review, and their applicability is noted in Table 1.

**Table 1.** The location of PV plants is considered for study in the literature.

Parameters	References									
	^14^	^15^	^16^	^17^	^18^	^19^	^20^	^21^	^22^	^23^
Grid connected	Yes	Yes	-	-	Yes	Yes	-	Yes	-	Yes
Rooftop	-	Yes	Yes	Yes	Yes	-	Yes	-	Yes	-
Residential areas	-	Yes	Yes	Yes	-	-	Yes	Yes	-	Yes
Lake and river	Yes	-	-	-	-	Yes	-	-	-	Yes
Farms	Yes	-	-	-	-	-	Yes	Yes	-	-

One of the first R analysis types thoroughly explored and applied realistically in many situations is linear regression. Creating a mathematical model that may be used to forecast one variable, known as the dependent variable, can be characterized using another variable, the independent variable. The degree of the linear relationship between two variables is measured using correlation analysis. This is because models with a linear dependence on their unknown parameters are more readily fitting than models with non-linear dependence because it is simpler to identify the data samples of the resulting estimators. ^24^

On the other hand, a significant amount of data must be managed, so the regression model is useful. ^25^ It is frequently used to predict time-series and regression models using conventional estimate approaches, which involve consideration of the predictor variables, the target variable, and their relationship. ^26^ This study compares the two models’ abilities to accurately forecast PV module performance: linear and non-linear regression models. A logarithmic linearized equivalent model serves as the mathematical representation of the non-linear model. In this paper, the site which has been selected is based on SRM Institute of Science and Technology in Kattankulathur, Chennai City, in Tamil Nadu, India. Many studies were conducted in our literature to investigate the behavior during one year, from January 2020 to December 2020. Performance parameters like global horizontal irradiation, energy yield, and capacity factor have been calculated. The power plants are installed on the rooftop of the Mechanical C Block, Civil Engineering Block, and Science & Humanities Block of SRM Institute of Science and Technology (SRMIST), Kattankulathur, 603203. In the paper, the description of installed PV systems and site details are discussed.

Further, the simulation of the grid and the calculated results are shown through tables and graphs. Towards the end of the paper, the economic factor and environmental impacts are discussed. Global solar radiation (GHI) of Asia and global solar radiation (GHI) of India are illustrated in Figure 1. These global solar radiation maps are downloaded from Solargis, where several collections of solar resource maps are available for research purposes. ^27^ Various case studies on the PV performance are accomplished by researchers by considering certain locations, ^28^^,^^29^ based on techno-economic assessment, ^30^^,^^31^ feasibility studies, ^32^^,^^33^ comparative studies, ^34^ optimizing the performance, ^35^ solar insolation studies and estimations ^36^^–^^38^ and several other factors. ^39^^–^^46^

## Methods

This section discusses the methodology followed to make this case study. Initially, the linear regression model is obtained using solar radiation, temperature, and wind speed data from NREL (National Renewable Energy Laboratory). Then, regression equations are obtained from this prediction model for further study. This statistical analysis will give the correlation among the control factors and its significance. The results from the prediction model will be compared with the running 52-kW plant installed in the institution. Finally, a complete description of all three 52-kW grid-connected PV systems is presented with the photographs, satellite map, and the specification of BoS (Balance of Solar PV system).

**Figure 2.**
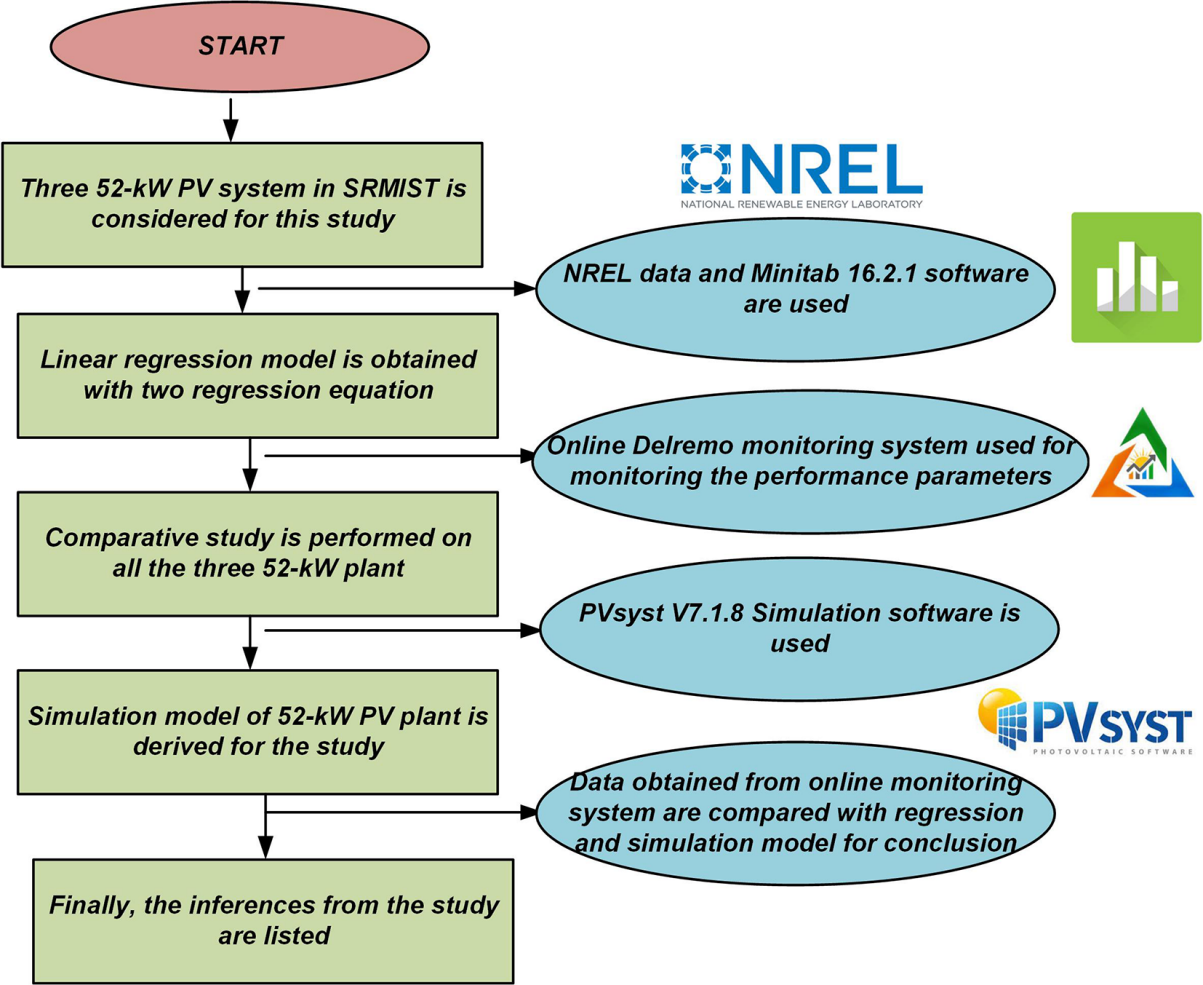
Methodology followed for the study.

After that, a comparative study is performed on all three 52-kW grid-connected PV systems with respect to energy yield, performance ratio, capacity utilization factor, C _O2_, and diesel saved. The procedure followed for this comparative analysis is presented in detail in a separate section. This 52-kW plant is simulated in PVsyst V7.1.8 simulation software and the results obtained are compared with the real-time data for 2020. This comparison will help us observe the performance of all three 52-kW PV systems. Finally, the inferences from the study are observed and listed for the conclusion. The flowchart of the methodology followed for the study is shown in Figure 2.

## Description of three 52-kW grid-connected PV system

**Figure 3.**
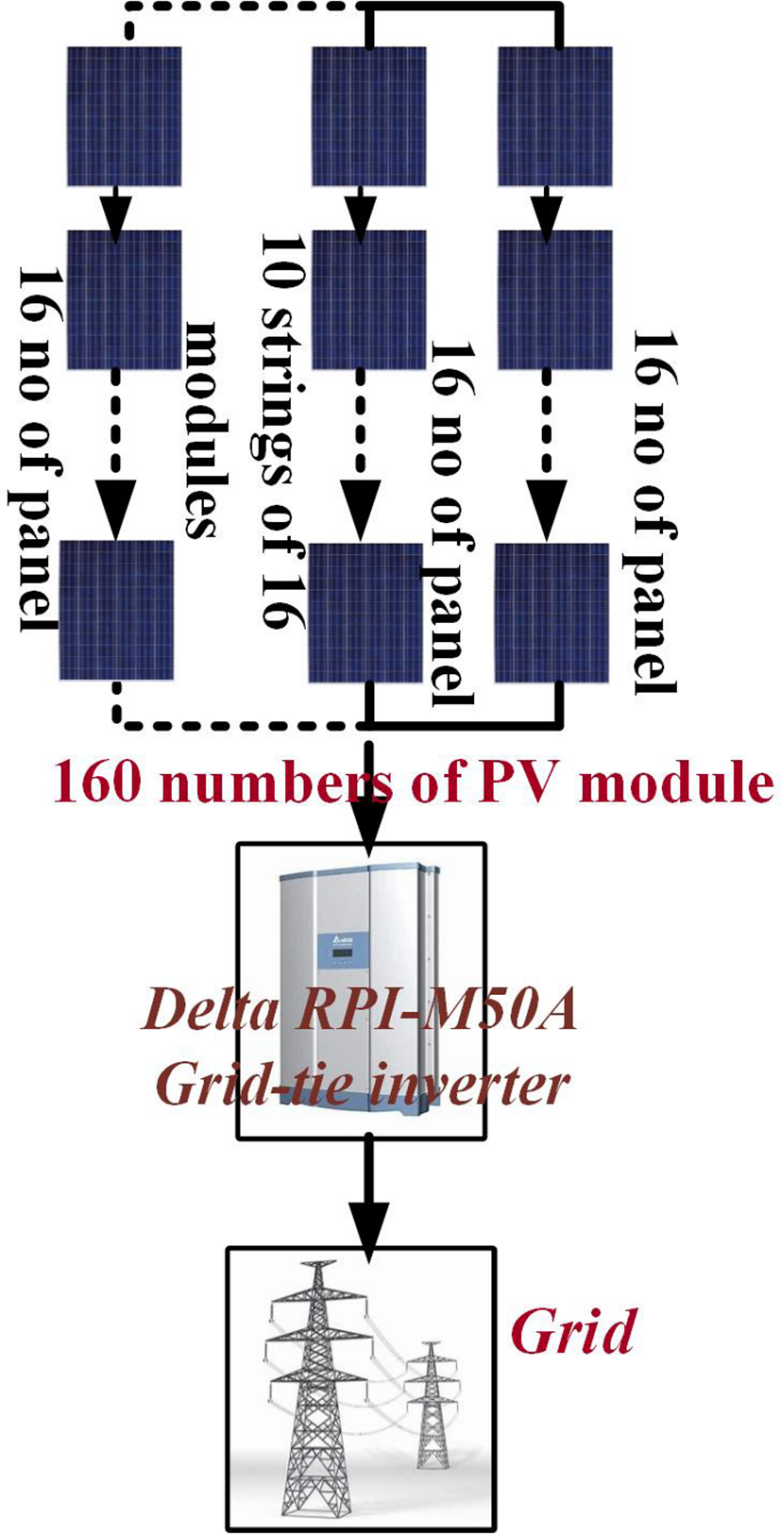
52-kW Solar PV plant.

The major components of the grid-connected PV system are a solar array, inverter with maximum power point tracking (MPPT), AC and DC disconnect, and other protective and connective equipment to the grid. It is more effective than a standalone PV system because it eliminates the losses incurred in energy storage. Another significant advantage of the grid-connected system is the eradication of the problem incurred due to the presence of batteries, i.e., cost and replacement. The general schematic diagram of all three 52-kW PV systems is represented in Figure 3.

### Site location

All three 52-kW solar power plants are located at SRMIST with latitude and longitude of 12.8231° N, 80.0442° E, and elevation above the sea level of 51 m. Since the generated PV power significantly depends on the sun’s position and its radiation intensity, the institute studied solar radiation for one year and opted for these three locations on the campus.

**Figure 4.**
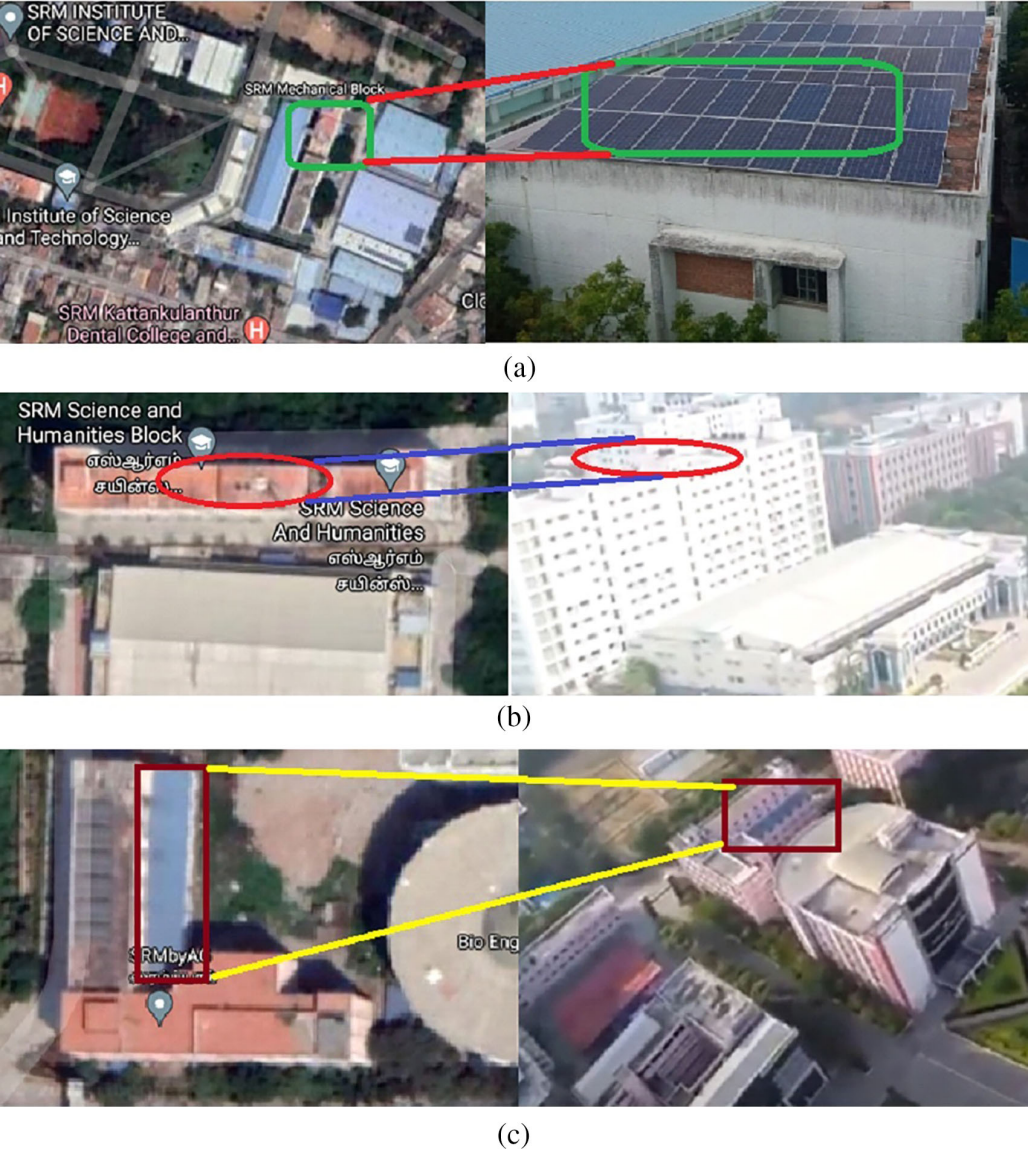
Site description (a) Mechanical 'C' block; (b) Science and Humanities; (c) Chemical Engineering block.

Figure 4(a)-(c) presents the description of all the sites taken for the study. In addition, satellite map images and photographs of the Mechanical ‘C’ block, Science and Humanities, and Chemical Engineering block are depicted in Figure 4(a), (b), and (c), respectively.

### Layout of PV plant

Three 52-kW PV plants occupy a rooftop area of 304 square meters. The plant is divided into ten strings with 16 panels in series. Each string has the capacity to generate 5.2 kW of power and the ten strings are combined to generate the power of 52 kW. All ten strings are connected to the main string combined box, which is connected to Delta RPI M50 A commercial inverter. All three 52-kW plants are installed with the structure as mentioned above. All these plants work with a central inverter system. The output of the plant is connected to the grid. The generated power is used for the lighting and other appliances in the institute.

### Tilt angle consideration for optimum utilization

Typically, in many solar plants, the tilt angle of the PV panels is made equal to the latitude of the geographical location of the PV plant. All three plants have fixed tilt angles, and the institute does not plan for any modern techniques to tilt the panel to produce efficient output. Since the latitude of the Kattankulathur location is 12.83°, the tilt angle of the three 52-kW solar PV plants is 13.3°.

### Solar panel specification

The rating of PV panels in the 52-kW plant is 325 W polycrystalline. It is a fixed type with a weight of 21.5 kg. The efficiency of the panel is 16.72%. The number of cells in this panel is 72 cells. This 325 W panel has a maximum voltage of 37.88 V and an open circuit voltage of 45.86 V. This polycrystalline PV panel’s maximum and short circuit current are 8.59 A and 9.06 A, respectively. To maintain the efficiency of the PV modules, the maintenance team regularly cleans the panels.

### Inverter specification

A 50 kVA inverter converts the DC power to AC power. The range of DC and AC voltage of the inverter are 200-1000 V and 320-480 V, respectively. The inverter’s efficiency is 98.60%, and the total input current is 100 A. The total harmonic distortion is less than 3%, with a 45-55 Hz frequency range. It has an inbuilt disconnect switch.

**Figure 5.**
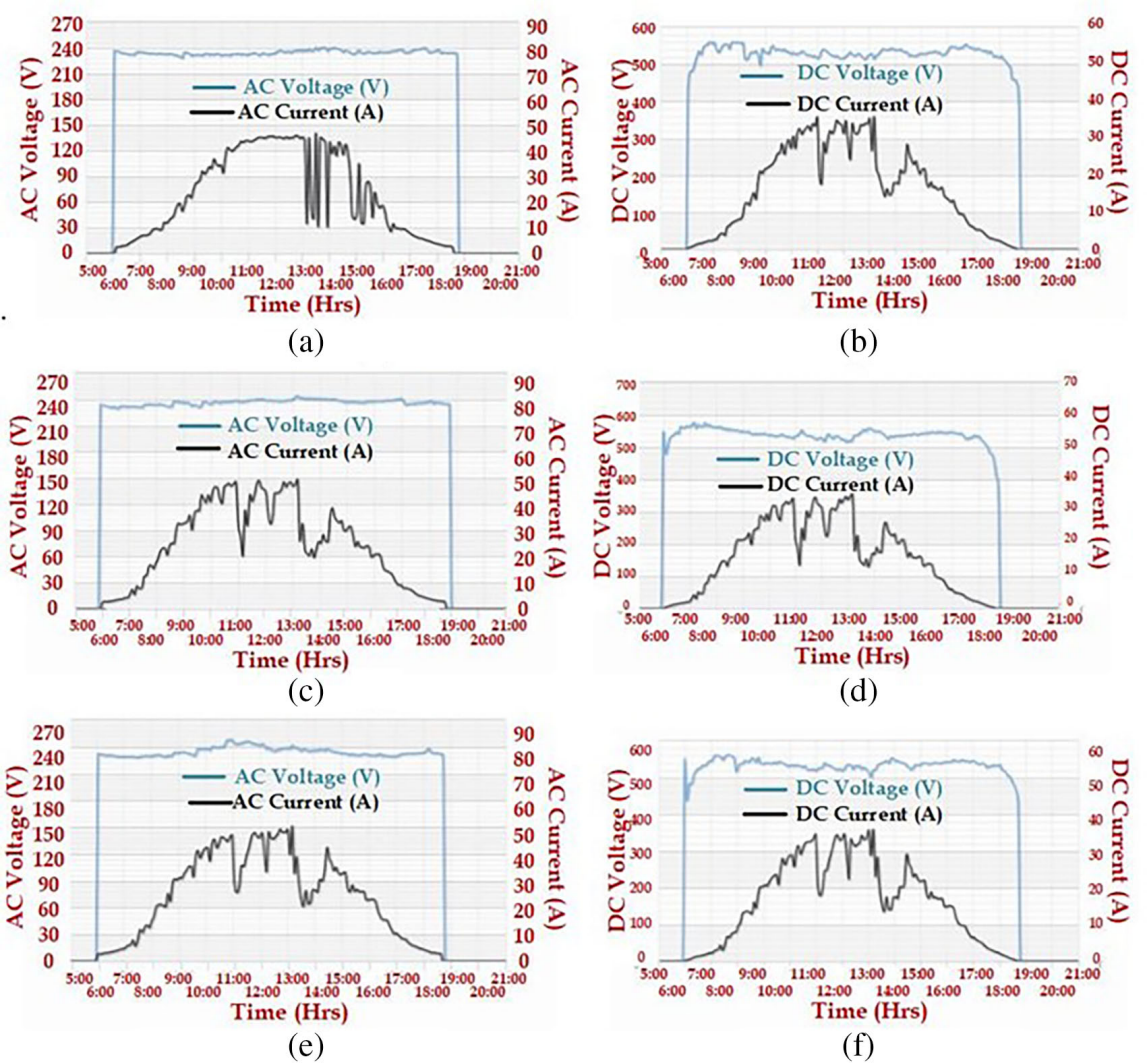
Voltage and current on June 15 ^th^, 2020 (a) Faculty of science and humanities: AC (b) Faculty of science and humanities: DC (c) Chemical Engineering lab: AC (d) Chemical Engineering lab: DC (e) Mechanical ‘C’ block: DC (f) Mechanical ‘C’ block: DC.

**Figure 6.**
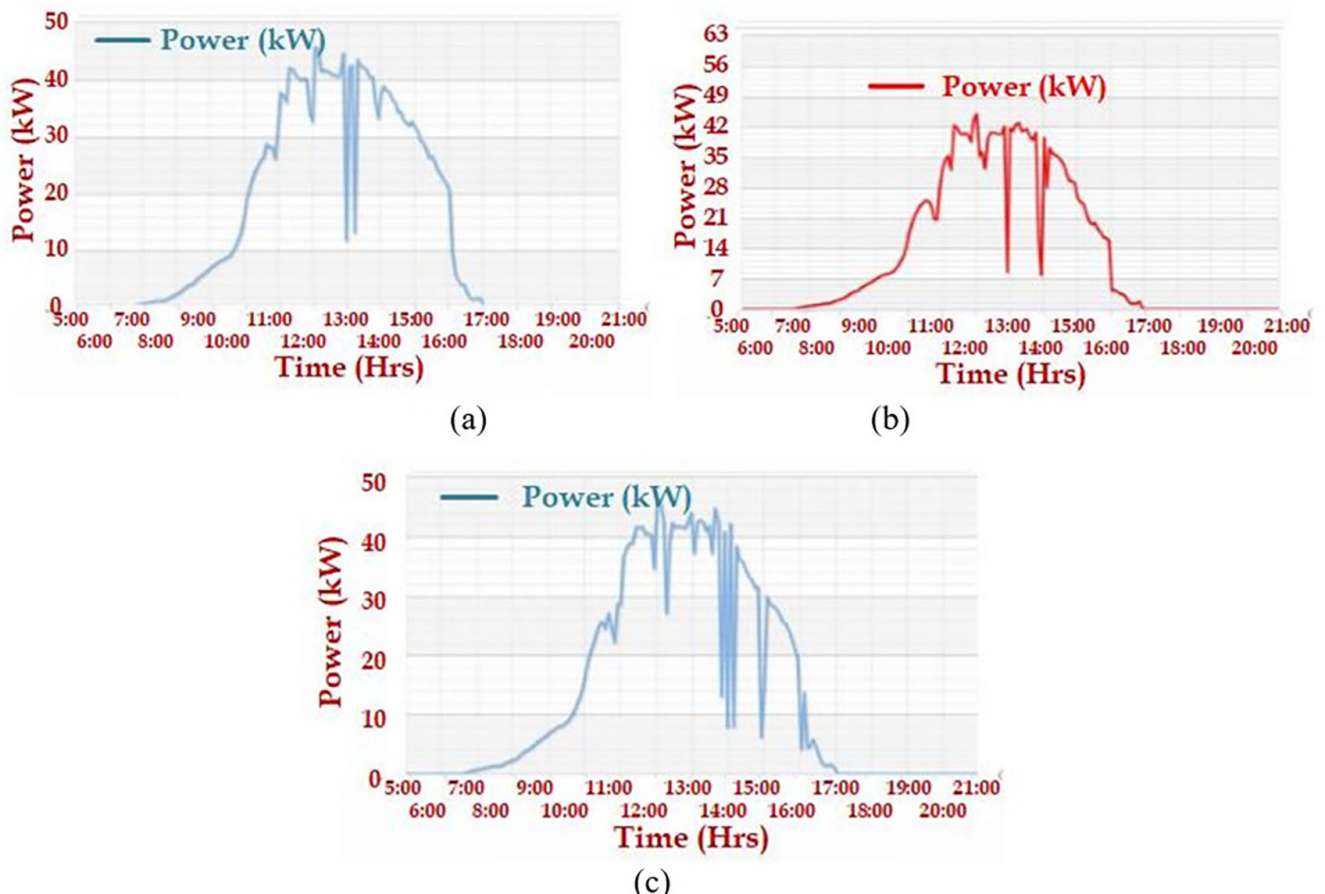
AC power on 22 ^nd^ October 2020 (a) Faculty of science and humanities; (b) Chemical Engineering lab; (c) Mechanical ‘C’ block.

To observe the DC and AC voltage, current, and power of the plant, a few graphs are presented in Figures 5 and 6. Figure 5(a)-(f) presents the AC and DC voltage and current of all the 52-kW power plants. This observation is drawn from June 15 ^th^, 2020. Similarly, the AC power output of three solar PV plants was observed on October 22 ^nd^, 2020; these graphs are presented in Figure 6. Figure 3(a)-(c) presents the AC power output of the 52-kW plant of the Faculty of Science and Humanities, Chemical Engineering lab, and mechanical ‘C’ block, respectively.

## Regression analysis for the selected site

The chosen site data was gathered on an hourly basis. The selected location receives 9 hours of solar radiation every day on average. For prediction, the AC hourly produced energy, direct beam and diffused radiation, ambient temperature, and wind velocity of the chosen site were considered. The regression model for the creation of AC energy outputs was developed using average hourly data at the location SRMIST, Kattankulathur, Tamil Nadu, India. From the prediction model, the regression equation was derived. Equation ((1), which has a linear relationship with beam radiation, diffused radiation, temperature, and wind speed, is used to estimate AC power from PV panels.

A regression model is a statistical method for determining the connection between the control variables. It is essential to check for residual plots before developing a regression equation to ensure linear regression. The statistical analysis and generation of the regression model for the system under consideration were carried out using Minitab software version 16.2.1.

ρ=4.188+0.030131α+0.042497β−0.18717γ+0.1261μ (1)

Where *ρ* - AC energy output (kWh), *α* - beam irradiance (W/m ^2^), *β* - diffuse irradiance (W/m ^2^), *γ* - ambient temperature (C), and *μ* - wind speed (m/s).

A graphical method of analyzing residuals is essential to assess for a ‘good fit’ regression model the best fit of a set of data in a regression line. The residual plots reveal the degree of correlation between the variables and the projected results. The figure shows the residual plots of AC energy yield. As demonstrated in the figure, the points are spaced linearly straight. It indicates that the projected and actual values are more closely related, which is denoted as a normal probability plot.

**Figure 7.**
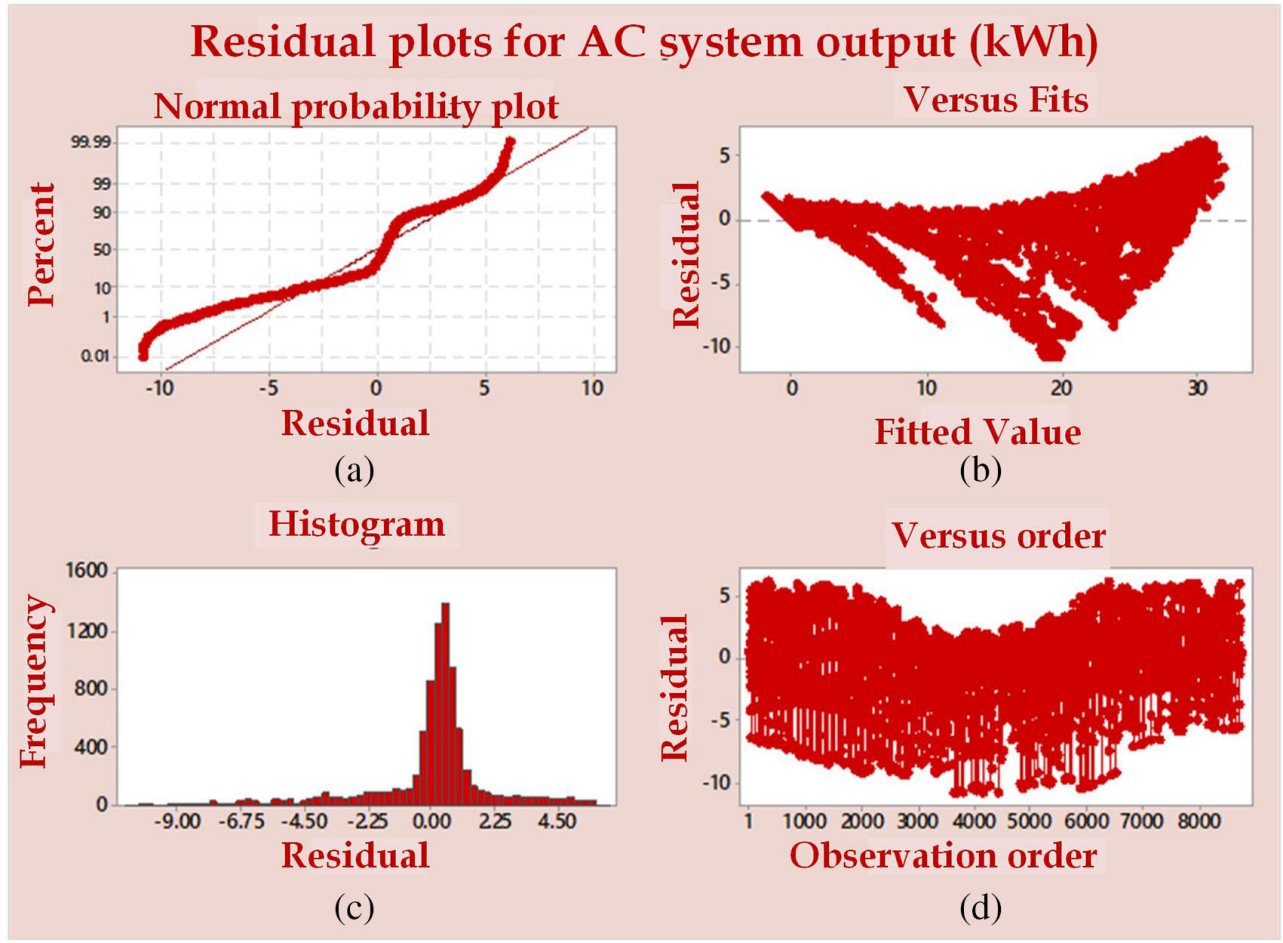
Residual plot of AC energy.

Figure 7 shows a comparison between residual and anticipated values. Both appear to be the most similar to one another, so there is very little difference between them. The histogram plot of AC energy is shown in Figure 7. In the histogram graph, clear data regarding the residuals are shown. The figure demonstrates the residuals vs. trial run order. Both positive and negative residual values are present, indicating the existence of certain relationships. The models show promise for adequacy due to the thorough study of AC residual plots.

**Figure 8.**
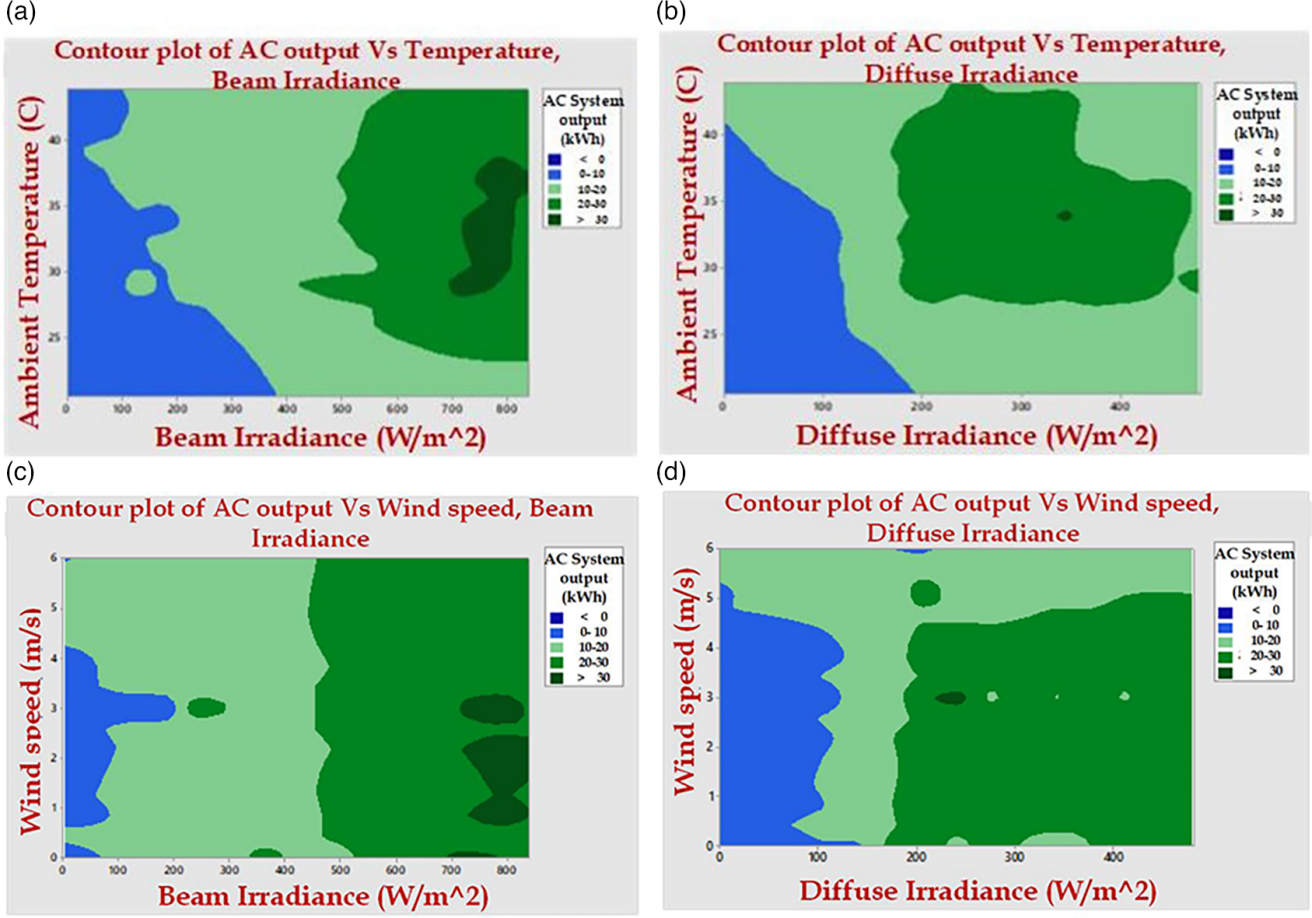
Influence of (a) Beam Irradiance and Temperature, (b) Diffuse Irradiance and Temperature, (c) Beam Irradiance and Wind Speed, (d) Diffuse Irradiance and Wind Speed.

The R ^2^ (Coefficient Determination) value of the generated regression model is higher, indicating appropriate accuracy. For AC energy, the R ^2^ value achieved is 96.39 percent. The corrected R ^2^ (R _adj_) value is 96.95 percent, indicating that the generated regression model is very significant. In addition, the R ^2^ (R _pred_) value obtained is 93.54 percent. Figure 8 depicts the influence of irradiance and temperature on the AC energy produced. At maximum beam irradiance and temperature median, the maximum array energy production is seen in Figure 8.

Increased temperature may result in a drop in production. The figure shows a high array output at the median of diffused radiation and temperature. It has been discovered that for the installed plant to produce more power, the temperature must be between low and high. The figure depicts the influence of irradiance and wind speed on the AC energy produced. The illustration depicts the effects of wind speed and beam irradiance on AC output shown in the image. For a high Voltage AC output, a full beam irradiance and a medium wind speed are required.

In the illustration, the median of the graph yields the highest output. As a result, it is found that maximum beam irradiance, medium dispersed radiation, temperature, and wind speed are the finest examples of high production yields.

## Procedure taken for the analysis

These 52-kW plants are analyzed, and their performance is studied by dividing the study into three stages.

***First stage:*** Retrieving data from the online (DelREMO) monitoring system of all three plants. The plant location and its structure are also studied thoroughly.

***Second stage***: The key metrics like yield ratio, performance ratio, and capacity utilization factor of the plants are analyzed and compared.

***Third stage:*** Finally, the energy yield of the plants is compared with the result obtained from the modelling software PVsyst 7.1.8. The loss diagram of the plant is obtained and discussed.

**Figure 9.**
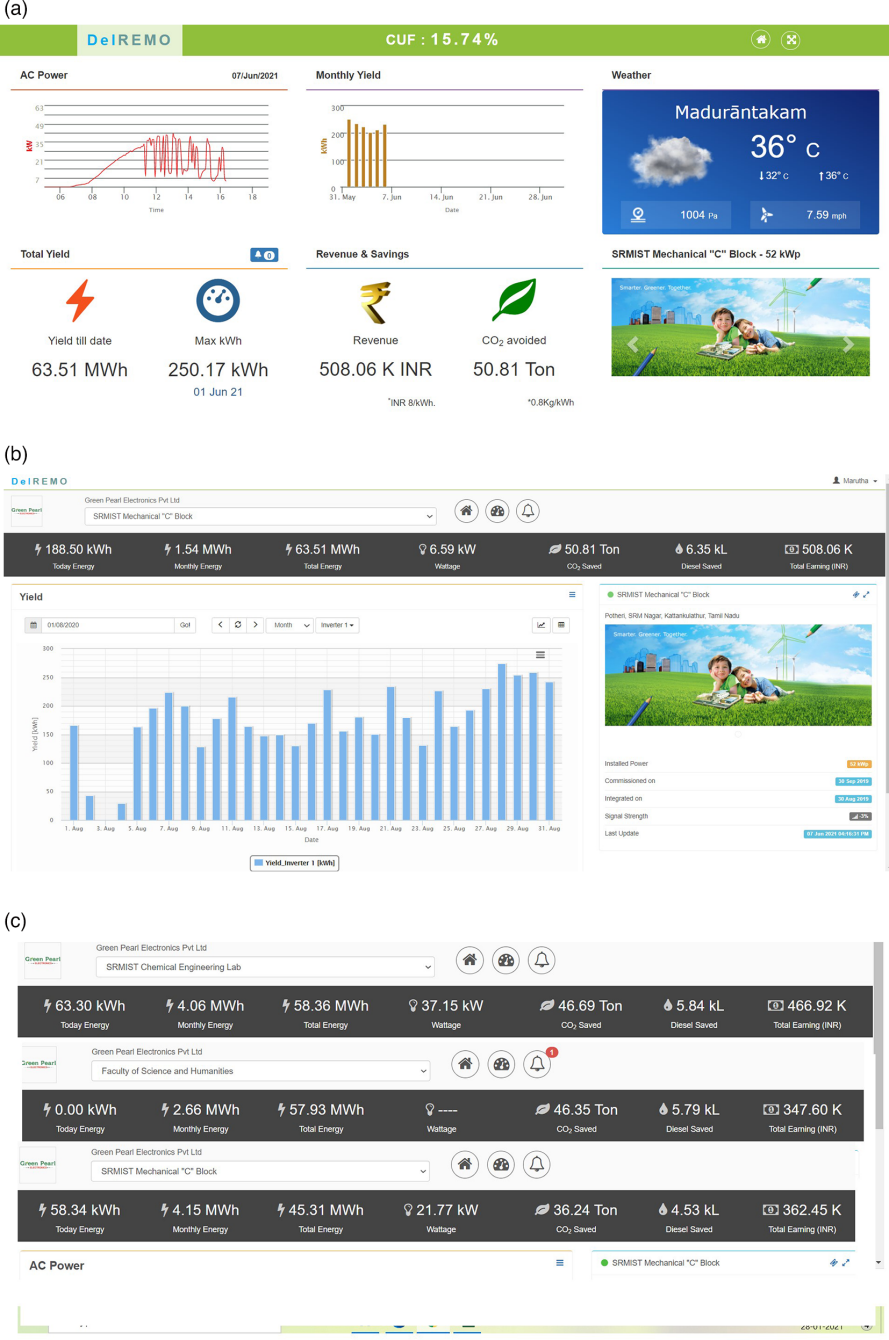
DelREMO online monitoring system (a) Home screen taken on June 7 ^th^ 2021; (b) AC energy yield display on August 1 ^st^, 2020, for Mechanical 'C' block 52 kW plant; (c) Comparative dashboard of all the three 52 kW PV plant.

**Figure 10.**
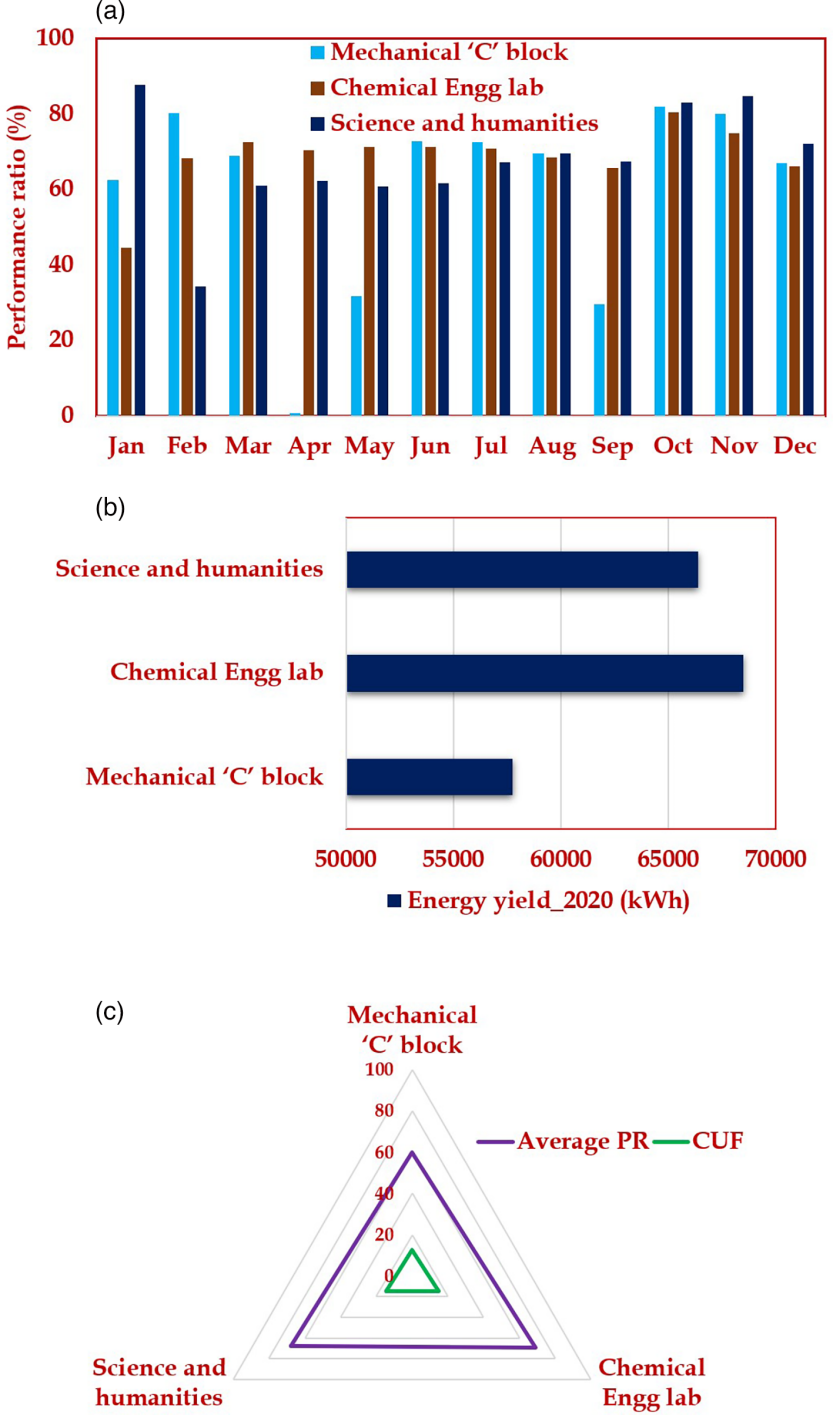
Comparison of all the three 52-kW PV plant (a) Performance ratio (PR); (b) Energy yield; (c) Average capacity utilization factor and PR.

There are specific performance parameters like reference yield, array yield, final yield, performance ratio and capacity utilization factor to determine the overall system’s performance. International Energy Agency has developed certain performance parameters for evaluating and analyzing the performance of grid-connected PV systems. ^9^ DelREMO online monitoring system is shown in Figure 9. Comparison of all three 52-kW PV plant is illustrated in Figure 10. Performance parameters of the 52-kW power plant at Mechanical ‘C’ block, chemical engineering block, Faculty of Science and Humanities is listed in Tables 2, 3 and 4, respectively. A comparison of key highlights of 52-kW power plants at the institute is given in Table 5.

**Table 2.** Performance parameters of the 52-kW power plant at Mechanical ‘C’ block.

Parameters	Jan	Feb	Mar	Apr	May	Jun	Jul	Aug	Sep	Oct	Nov	Dec
**Global horizontal irradiation (kWh/m** ^**2**^ **/mth)**	153	164	193	190	187	162	158	161	157	139	120	130
**Temperature (°C)**	25	26.1	28	29.7	31.6	30.7	30.5	29.6	28.8	27.5	25.5	24.9
**AC Energy yield (kWh)**	4975	6845	6928	81	3097	6146	5964	5840	2428	5936	4990	4545
**Reference yield**	153	164	193	190	187	162	158	161	157	139	120	130
**Final yield**	95.7	131.6	133.2	1.6	59.6	118.2	114.7	112.3	46.7	114.2	95.9	87.4
**Performance ratio (%)**	62.5	80.2	69	0.84	31.8	72.9	72.6	69.7	29.7	82	80	67
**Capacity factor (%)**	13.3	18.3	18.5	0.22	8.3	16.4	15.9	15.6	6.5	15.8	13.3	12.1

**Table 3.** Performance parameters of the 52-kW power plant at Chemical Engineering lab.

Parameters	Jan	Feb	Mar	Apr	May	Jun	Jul	Aug	Sep	Oct	Nov	Dec
**Global horizontal irradiation (kWh/m** ^**2**^ **/mth)**	153	164	193	190	187	162	158	161	157	139	120	130
**Temperature (°C)**	25	26.1	28	29.7	31.6	30.7	30.5	29.6	28.8	27.5	25.5	24.9
**AC Energy yield (kWh)**	3543	5855	7273	6977	6943	6006	5815	5745	5376	5828	4657	4479
**Reference yield**	153	164	193	190	187	162	158	161	157	139	120	130
**Final yield**	68.1	112.6	139.9	134.2	133.5	115.5	111.8	110.5	103.4	112.1	90	86.1
**Performance ratio (%)**	44.5	68.3	72.5	70.5	71.3	71.3	70.8	68.6	65.8	80.5	75	66.2
**Capacity factor (%)**	9.5	15.6	19.4	18.6	18.5	16.0	15.5	15.3	14.4	15.6	12.5	11.9

**Table 4.** Performance parameters of the 52-kW power plant at Faculty of Science and Humanities.

Parameters	Jan	Feb	Mar	Apr	May	Jun	Jul	Aug	Sep	Oct	Nov	Dec
**Global horizontal irradiation (kWh/m** ^**2**^ **/mth)**	153	164	193	190	187	162	158	161	157	139	120	130
**Temperature (°C)**	25	26.1	28	29.7	31.6	30.7	30.5	29.6	28.8	27.5	25.5	24.9
**AC Energy yield (kWh)**	6989	2925	6167	6162	5910	5191	5523	5821	5515	5996	5287	4886
**Reference yield**	153	164	193	190	187	162	158	161	157	139	120	130
**Final yield**	134.4	56.3	118.6	118.5	113.7	99.8	106.2	111.9	106.1	115.3	101.7	93.9
**Performance ratio (%)**	87.8	34.3	61.1	62.3	60.8	61.6	67.2	69.5	67.5	83	84.7	72.2
**Capacity factor (%)**	18.7	7.8	16.5	16.5	15.8	13.8	14.8	15.5	14.7	16.0	14.1	13.0

**Table 5.** Comparison of key highlights of 52-kW power plants at the institute.

Solar PV plant	Date of commencement	Co2 saved (Ton)	Diesel saved (kL)	Total Earning (INR)
Mechanical 'C' block	November 20th 2019	38.4	4.8	384.3 K
Chemical Engg lab	September 25th 2019	48.9	6.1	488.9 K
Science and humanities	September 30th 2019	46.4	5.9	347.6 K

## Simulation using PVsyst

The maximum energy generated is in the month of March (6627 kWh), and the minimum is generated during July (4428kWh). The total energy produced during that year was 64606 kWh.

### Balances and main results

As shown in Table 6, the annual global irradiation is 1913.8 kWh/m ^2^. The total energy obtained is 66903 kW/h. The average ambient temperature is 28.17°C and obtained annual average performance ratio obtained is 88.1%. A comparison of monitored results with the results acquitted from PVsyst V7.1.8 is listed in Table 7.

**Table 6.** Results obtained from PVsyst simulation.

Month	GHI kWh/m ^2^	DHI kWh/m ^2^	Temp °C	Glob-Inc kWh/m ^2^	Glob-Eff kWh/m ^2^	E_Array kWh	E_Grid kWh	PR ratio
January	153.5	66.47	24.97	186.60	183.00	6688	6461	0.89
February	164.5	62.04	26.13	186.80	182.90	6555	6333	0.87
March	193.2	75.73	28.04	196.00	190.90	6861	6627	0.87
April	190.3	83.80	29.66	172.50	166.90	6068	5860	0.87
May	187.3	88.00	31.67	154.80	148.50	5428	5242	0.87
June	162.7	90.30	30.71	131.10	125.20	4649	4490	0.88
July	157.7	88.07	30.46	129.60	123.90	4588	4428	0.88
August	161.2	93.98	29.62	142.50	137.50	5082	4907	0.88
September	156.8	81.87	28.80	151.40	146.90	5375	5188	0.88
October	138.8	78.86	27.52	144.60	141.10	5170	4990	0.89
November	119.5	70,99	25.55	134.30	131.20	4866	4699	0.90
December	128.3	67.39	24.80	153.70	150.60	5572	5382	0.90
**Year**	**1913.8**	**947.50**	**28.16**	**1883.90**	**1828.60**	**66902**	**64607**	**0.88**

**Table 7.** Comparison of monitored results with the results acquitted from PVsyst V7.1.8.

Serial number	Month (2020)	PVsyst V7.1.8 result (kWh)	Online monitored result of 52-kW plant (kWh)		
			Mechanical ‘C’ block	Chemical Engineering	Science and Humanities
1	January	6688	4975	3543	6989
2	February	6555	6845	5855	2925
3	March	6861	6828	7273	6167
4	April	6068	81	6977	6162
5	May	5428	3097	6943	5910
6	June	4649	6146	6006	5191
7	July	4588	5964	5815	5523
8	August	5082	5840	5745	5821
9	September	5375	2428	5376	5515
10	October	5170	5936	5828	5996
11	November	4866	4990	4657	5287
12	December	5572	4545	4479	4886
	Total	66903	57675	68497	66372

### Normalized production

**Figure 11.**
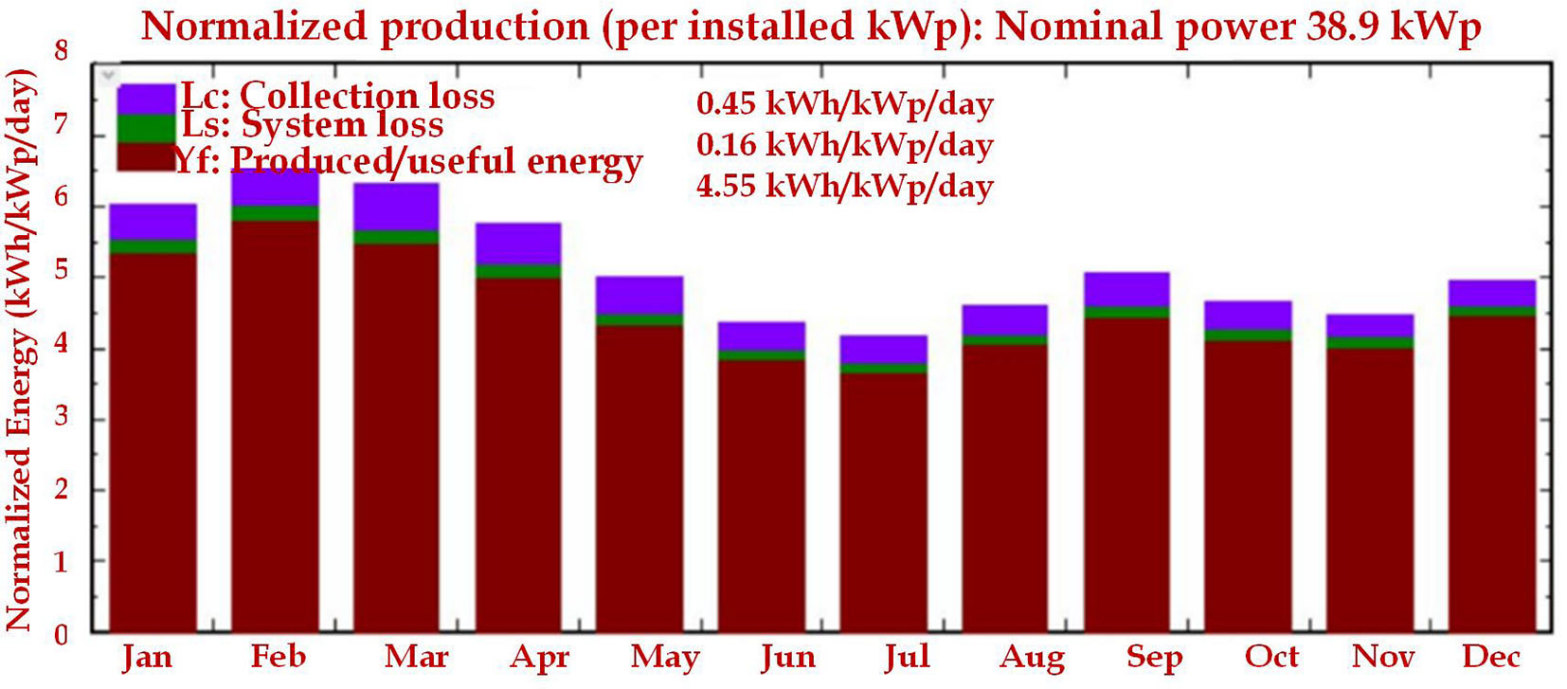
Normalized production of 52-kW PV plant.

Figure 11 shows the L _c_ value recorded as 0.45kWh/kWp/day and the L _a_ value as 0.16kW/kWp/day. Similarly, Y _F_ is recorded as 4.55kWh/kWp/day.

### Loss diagram

**Figure 12.**
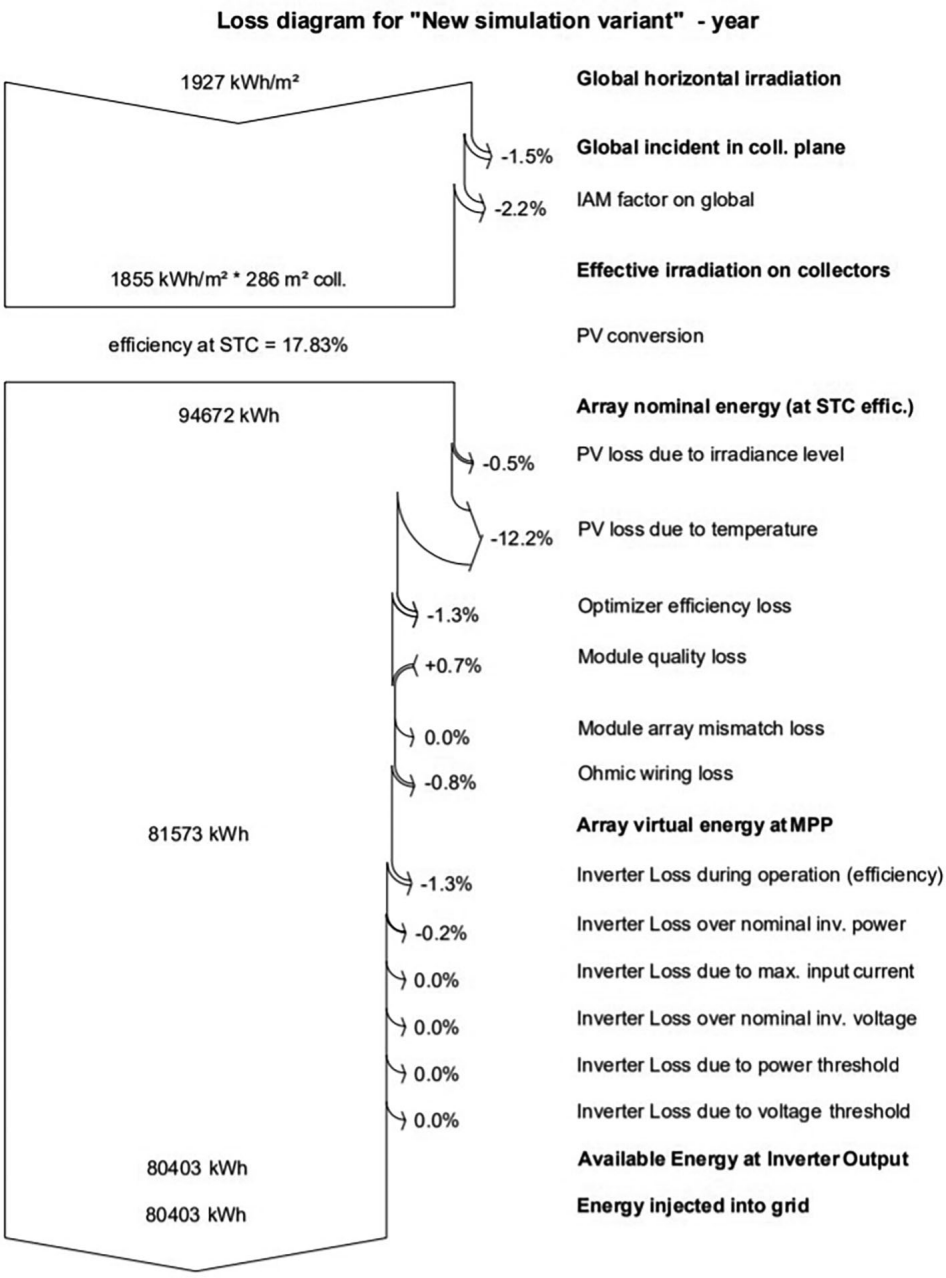
Loss diagram of 52-kW PV plant.

The global horizontal irradiance is 1927 kWh/m ^2^/y, as shown in Figure 12. The effective irradiation on the collector plane is 1842 kWh/m ^2^/y. After the PV conversion, the nominal array energy is 5559 kWh. The efficiency of the PV array is 15.46% at STC, while the virtual energy is 4726 kWh. The energy at the output after having the inverter losses comes out to be 4524 kWh.

### Inference from the study

The results obtained from the online monitoring system (DelREMO) is compared with the data acquired from the linear regression model and PVsyst software. From Table 5, the following observations are made:

• The actual performance 52-kW plant in Science and Humanities closely matches the results obtained from the PVsyst.
• The energy yield of the 52-kW plant in Chemical Engineering is slightly higher compared with the results obtained from the PVsyst.
• The 52-kW plant in the Mechanical ‘C’ block operates with underperformance compared with the results obtained from the PVsyst and the other two PV plants.
• The energy yield of the 52-kW plant in Chemical Engineering is high from March to July.
• The energy yield of the 52-kW plant in Science and Humanities is high from August to December.
• The Science and Humanities building is located in a place without any hindrance caused by tall buildings and trees, whereas the plant on the Mechanical ‘C’ block is surrounded by many tall buildings adjacent to the location.

## Conclusions

A performance study of three 52-kWgrid connected solar photovoltaic power plants installed on the Mechanical Engineering Block, Chemical Engineering Block, and Science & Humanities Block of rooftop SRMIST Kattankulathur was evaluated on an annual basis. The observations drawn from this case study are:

• The maximum energy yield was observed in February (6828 kWh), whereas the lowest was recorded in May (81 kWh) from the Mechanical ‘C’ block.
• The maximum energy yield was observed in March (7273 kWh), whereas the lowest was recorded in January (3543 kWh) from the Chemical Engineering block.
• The maximum energy yield was observed in January (6989 kWh), whereas the lowest was recorded in February (2925 kWh) from Science and Humanities block.
• By comparing the energy yield of the three 52-kW power plants, it is noted that the month of the maximum and minimum of the three plants are not similar. However, the energy yield value is nearer for the solar plants except for the Mechanical ‘C’ block.
• The lowest energy yield in May in the Mechanical ‘C’ block is due to the plant’s shutdown during the lockdown during the pandemic covid situation.
• The comparison of measured energy yield with PVsyst divulges that the 52-kW solar plant is functioning closer to the forecasted generation of energy yield from the PVsyst 7.1.8.
• This case study gives an insight into identifying the location for large-scale implementation of PV plants across India. Also, this study helps calculate and evaluate other operational data based on net energy output. The obtained data on the PV system can also be helpful in large-scale applications.
• All the operating plants have a good PR ratio. Also, the plant has been operating and feeding energy to the grid at a good percentage.
• A precise forecast of solar energy generated is critical in today’s scenario for a specific site. This article may help researchers to have an insight understanding of forecasting AC power produced by the installed power plants.

## Data Availability

OSF: Solar data, https://doi.org/10.17605/OSF.IO/NCRDU. ^47^ This project contains the following underlying data:
• AC power 1st May 2020.xlsx2023-06-14 11:38 AM • AC voltage and current 1st May 2020.xlsx2023-06-14 11:38 AM • Solar On Grid power plant monthly Generation_2020-Specific data.xlsx Data are available under the terms of the Creative Commons Attribution 4.0 International license (CC-BY 4.0).
