# Supplementary material for: Performance evaluation and comparative study of three 52-kW PV plants in India: a case study
Source: F1000Res. 2023 Aug 31;12:1068. [Version 1] doi: 10.12688/f1000research.134731.1 (PMC11200062; doi:10.12688/f1000research.134731.1)
Supplement: Peer review history

# Reviewer report: Reviewer response for version 1

**DOI:** 10.5256/f1000research.147811.r202435

**Published:** 2024-06-25

**Authors:** Zahira R.

**Affiliations:** 1 Associate Professor, Department of Electrical and Electronics Engineering, B.S. Abdur Rahman Crescent Institute of Science and Technology, Chennai, Tamil Nadu, India

The manuscript content is very well written. The prediction model for the 52-kW power plant is analyzed using PV syst software and the data's are compared well with the different scenario in institution buildings. In addition the model-based prediction equations are derived using the Minitab 16.2.1 software, and the results are compared with the real-time AC energy yield acquired from the three 52-kW plants for the year 2020.

Is the case presented with sufficient detail to be useful for teaching or other practitioners?

Yes

Is the work clearly and accurately presented and does it cite the current literature?

Yes

If applicable, is the statistical analysis and its interpretation appropriate?

Yes

Are all the source data underlying the results available to ensure full reproducibility?

Yes

Are the conclusions drawn adequately supported by the results?

Yes

Is the background of the case’s history and progression described in sufficient detail?

Yes

Reviewer Expertise:

microgrid, power quality, renewable energy

I confirm that I have read this submission and believe that I have an appropriate level of expertise to confirm that it is of an acceptable scientific standard.

# Reviewer report: Reviewer response for version 1

**DOI:** 10.5256/f1000research.147811.r202434

**Published:** 2023-12-12

**Authors:** Deepa Kaliyaperumal

**Affiliations:** 1 Department of Electrical and Electronics Engineering, Amrita School of Engineering, Bengaluru, Karnataka, India

Work has been reported professionally with good technical analysis. the analysis is excellent.

 1)data availability to the user has to be mentioned in the content.

 2)the conclusion of before pandemic, during the pandemic and after the pandemic can be listed in a table

 3) The conclusion for summer and winter seasons can be listed

Is the case presented with sufficient detail to be useful for teaching or other practitioners?

Yes

Is the work clearly and accurately presented and does it cite the current literature?

Yes

If applicable, is the statistical analysis and its interpretation appropriate?

Yes

Are all the source data underlying the results available to ensure full reproducibility?

Partly

Are the conclusions drawn adequately supported by the results?

Yes

Is the background of the case’s history and progression described in sufficient detail?

Yes

Reviewer Expertise:

Renewable Energy technologies, Power electronics converters for application in Renewable, Electric Vehicle, Power Quality ...etc, Machine learniong and deep learning applications in all fields

I confirm that I have read this submission and believe that I have an appropriate level of expertise to confirm that it is of an acceptable scientific standard.

# Response

**Published:** 2023-12-13

**Authors:** Divya Navamani J

1)data availability to the user has to be mentioned in the content.

 Data availability-Underlying data

 OSF: Solar data, https://doi.org/10.17605/OSF.IO/NCRDU. 47

 It is mentioned in the article.

 2)the conclusion of before pandemic, during the pandemic and after the pandemic can be listed in a table.

 The plants were commissioned by Nov 2019. So, before pandemic, we took data from Jan 2020 -March 2020 and analysed in the article.

 3) The conclusion for summer and winter seasons can be listed

 Entire analysis is carried out for the year 2020 (Jan-Dec) for both summer and winter season, The PR for both the seasons are presented.

# Reviewer report: Reviewer response for version 1

**DOI:** 10.5256/f1000research.147811.r202437

**Published:** 2023-11-24

**Authors:** Arvind R. Singh

**Affiliations:** 1 Department of Electrical, Electronic and Computer Engineering, University of Pretoria, Pretoria, Gauteng, South Africa

1. What insights or suggestions may be obtained from the loss analysis conducted using PVsyst software to identify the elements contributing to the performance gap in the 52-kW photovoltaic (PV) plants? Furthermore, how can these observations be utilised to develop methods aimed at optimising the effectiveness and productivity of grid-connected photovoltaic (PV) systems operating in comparable environmental circumstances?

 2. What specific challenges or issues are associated with this rapid growth, and how does the research aim to address them in the context of performance evaluations of the 52-kW PV plants?

 3. This study offers a comprehensive analysis of the advantages and limits associated with different models. By examining these factors, we may get valuable insights into how the selection of a certain model type may influence the accuracy of photovoltaic (PV) performance forecasts.

 4. Can you elaborate on the practical applications and implications of the insights gained from this case study in terms of identifying suitable locations for large-scale PV plant implementations and the forecasting of solar energy generation in India, especially in the context of sustainable energy planning and grid integration?

Is the case presented with sufficient detail to be useful for teaching or other practitioners?

Partly

Is the work clearly and accurately presented and does it cite the current literature?

Yes

If applicable, is the statistical analysis and its interpretation appropriate?

Partly

Are all the source data underlying the results available to ensure full reproducibility?

Yes

Are the conclusions drawn adequately supported by the results?

Partly

Is the background of the case’s history and progression described in sufficient detail?

Yes

Reviewer Expertise:

NA

I confirm that I have read this submission and believe that I have an appropriate level of expertise to confirm that it is of an acceptable scientific standard, however I have significant reservations, as outlined above.

# Response

**Published:** 2023-11-27

**Authors:** Divya Navamani J

**Rebuttal letter**

 1. What insights or suggestions may be obtained from the loss analysis conducted using PVsyst software to identify the elements contributing to the performance gap in the 52-kW photovoltaic (PV) plants? Furthermore, how can these observations be utilised to develop methods aimed at optimising the effectiveness and productivity of grid-connected photovoltaic (PV) systems operating in comparable environmental circumstances?

**The losses can be obtained for the whole year or month-wise from PVsyst software. This loss diagram gives an insight into the seasonal effect and is used to evaluate the impact of different losses. Furthermore, the loss diagram, which shows the primary causes of losses, offers a quick and insightful look at the quality of a PV system design. In the presented article, we have given the losses incurred in the plant. As a future scope, we have planned to suggest an optimizing methodology to work on the productivity of grid-connected photovoltaic (PV) systems based on the losses incurred.**

**In addition, further studies can be made to increase the efficiency of PV systems by reducing the loss.**

 2. What specific challenges or issues are associated with this rapid growth, and how does the research aim to address them in the context of performance evaluations of the 52-kW PV plants?

**This article compares the performance evaluation of three PV plants with a 52-kW rating. The two plants are close to each other. The mechanical ‘C’ block plant is 1.3 km from the other two plants. The PR ratio of the Mechanical ‘C’ block plant is 60 %, whereas the other two plants are 68-69 %. All three plants were commissioned in 2019. The observations are made and informed the maintenance engineer after this case study.**

 3. This study offers a comprehensive analysis of the advantages and limits associated with different models. By examining these factors, we may get valuable insights into how the selection of a certain model type may influence the accuracy of photovoltaic (PV) performance forecasts.

**Yes. This case study is carried out to perform a comprehensive analysis of three 52-kW plants by theoretical study and using PVsyst software. Inference from the survey is drawn and presented in the manuscript.**

 4. Can you elaborate on the practical applications and implications of the insights gained from this case study in terms of identifying suitable locations for large-scale PV plant implementations and the forecasting of solar energy generation in India, especially in the context of sustainable energy planning and grid integration?

**This study helps to calculate and evaluate other operational data based on net energy output. The obtained data on the PV system can also be helpful in large-scale applications. The global horizontal solar irradiation of the location is presented with PR and CUF calculations. This will help us go for a large-scale solar PV plant on a site with a similar GHI.**

# Response

**Published:** 2024-04-15

**Authors:** Dr. Arvind R. Singh

The revision is satisfactory
